# Application study of multidisciplinary collaboration (MDT) integrated management model in perioperative management of patients with infectious nonunion

**DOI:** 10.3389/fsurg.2024.1335157

**Published:** 2024-02-28

**Authors:** Qing Lu, Zhaodong Bi, Yunxu Tian, Yuqing Li, Shanshan Zhang, Xiuting Li, Wenzhao Xing

**Affiliations:** ^1^ The Orthopedics and Traumatology Department, The Third Hospital of Hebei Medical University, Shijiazhuang, Hebei, China; ^2^ Faculty of Biological Sciences, University of Leeds, Leeds, United Kingdom; ^3^ Hebei Bone Research Institute, Key Laboratory of Biomechanics of Hebei Province, Shijiazhuang, Hebei, China

**Keywords:** multidisciplinary collaboration (MDT) integrated, management model, perioperative management, infectious nonunion, MDT intervention team

## Abstract

**Objective:**

To explore the effectiveness of a multidisciplinary treatment (MDT) integrated intervention model in the perioperative management of patients with infectious nonunion.

**Methods:**

80 patients with infectious bone defects treated in our hospital from January 2020 to January 2023 were selected. They were classified into MDT-integrated perioperative group (study group) and conventional control group according to the different management patterns, with 40 cases each. The incidence of wound infection, pin tract infection, delayed bone healing, deep vein thrombosis (DVT), joint stiffness, and nutritional indicators were compared between the two groups.

**Results:**

The rates of wound infection (*P* = 0.042), pin tract infection of Grade II or above (*P* = 0.006), delayed bone healing (*P* = 0.006), DVT (*P* = 0.033), and joint stiffness (*P* = 0.023) in the MDT integrated perioperative (study) group were significantly lower than those in the conventional care group (*P* < 0.05). With the extension of intervention time, the changes in body weight, levels of serum albumin (ALB), pre-albumin (PA), hemoglobin (Hb), and serum sodium (Na) in the study group were higher than those in the conventional care group (*P* < 0.05).

**Conclusion:**

The application of the MDT integrated intervention model in the perioperative period of patients with infectious nonunion is beneficial in reducing the risks of wound infection and pin tract infection of Grade II or above, lowering the incidence rates of lower limb DVT and joint stiffness, and reducing the risk of malnutrition, demonstrating high clinical application value.

## 1 Introduction

The incidence of post-traumatic bone infection has been showing an increasing trend year by year, which may be related to the increase in the number of open fractures and the usage rate of internal fixations. Addressing this issue is a major challenge faced by medical professionals today. Recent research data shows that the incidence of this disease varies from 0.4% to 16.1%, averaging about 5% (1). Among these, the infection rate post internal fixation of closed fractures is 1%, while for open fractures, it exceeds 15% (2), and can even reach 30% (3), with the highest being up to 55% (4). The infection rates post internal fixation of fractures at different sites vary slightly, with the rate for proximal tibial fractures being between 2.1% and 11.1%, averaging 6.9% (4, 5, 6), and for ankle fractures being between 1.1% and 6.1%, averaging 4.1% (7, 8). Additionally, geographical location and climate conditions also affect the incidence of bone infection (9).

**Figure 1:**
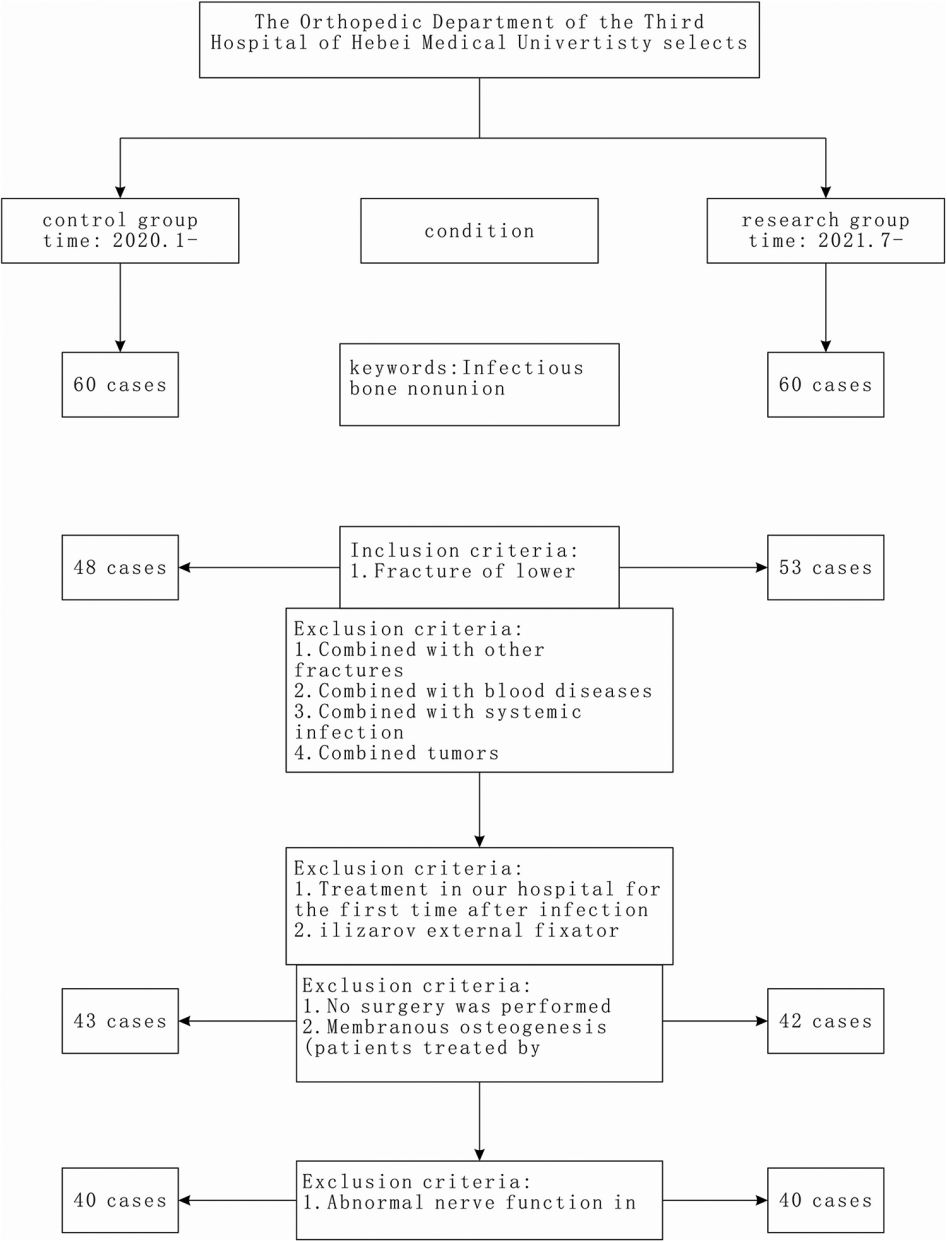
Case selection process.

**Figure 2:**
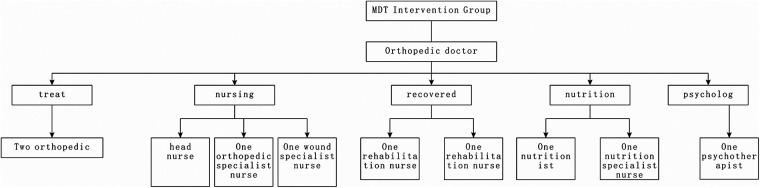
The composition of MDT intervention member.

Currently, patients with infectious nonunion might face various complications in treatment, such as non-healing at the osteotomy site, infection in the bone-lengthening area, poor osteogenesis in the regeneration area, and joint stiffness and contracture (10, 11). The prevention of such complications requires the intervention of multidisciplinary personnel. The multidisciplinary collaboration (MDT) integrated intervention model, which combines the treatment and care methods and knowledge of professionals from different specialties to comprehensively assess patients and provide individualized interventions, has been applied in patients with severe conditions and achieved significant results. However, it has not yet been applied to patients with bone infections. Therefore, this study applies the MDT integrated intervention model to patients with infectious nonunion, and the results are reported as follows.

## 2 Materials and methods

### 2.1 General information

All case data were derived from the Orthopedic Case Database of the Third Hospital of Hebei Medical University. We retrospectively screened 80 patients with infectious osteomyelitis treated from January 2020 to January 2023. This study was approved by the Ethics Committee of the Third Hospital of Hebei Medical University, with the ethical project number: W2020-024-1. All cases followed informed consent procedures.

Inclusion criteria: (1) Patients with lower limb fractures were selected; (2) Diagnosed with infectious nonunion according to the following standards and undergoing their first debridement and external fixation surgery in our hospital after nonunion; (3) Patient age ≥18 years; (4) Complete data for all items.

Diagnostic criteria for infectious nonunion: Infection at the fracture site affects the normal healing of the fracture. If the healing of the fracture shows no progress and cannot heal without intervention, it can be determined as nonunion.

Exclusion criteria: (1) Patients with pre-existing hematological diseases or severe systemic infection state before admission; (2) Patients with malignant tumors or significant organ dysfunction; (3) Patients with pre-existing lower limb neurological abnormalities before bone infection injury; (4) Patients with fractures in other locations; (5) Patients with psychiatric disorders; (6) Patients who did not undergo surgery did not receive surgery or were treated with the Masquelet technique; (7) Patients who did not complete data collection (Figure 1).

Eighty patients with infectious nonunion admitted to our hospital from January 2020 to January 2023 were selected as the subjects for this study. Based on the grouping by date, the subjects were divided into two groups: a standard care group (control group) with 40 cases and an MDT (Multidisciplinary Team) perioperative integrated intervention group (study group) with 40 cases.

### 2.2 Intervention methods

#### 2.2.1 Both groups of patients received perioperative interventions, including: patient admission health education, psychological counseling, routine preoperative and postoperative observation and management, nutritional dietary guidance, guidance on limb function exercises, medication guidance, discharge guidance, and other measures

#### 2.2.2 Responsible nurses and attending physicians primarily managed the control group

#### 2.2.3 The observation group established a multidisciplinary collaborative, integrated management of patients during the perioperative period, with doctors from various disciplines such as orthopedics (attending physician), nutrition department, rehabilitation department, and nurses assisting

The specific steps of the observation group’s MDT management mode are as follows (Figure 2):

(1) Formation of MDT Intervention Team: The team is mainly led by the head orthopedic surgeon and the chief nurse of the orthopedic department. The team members include professionals from various disciplines, such as treatment, nursing, rehabilitation, psychology, and nutrition. It comprises one head nurse, three responsible nurses, two orthopedic doctors, one rehabilitation therapist, one psychological counselor, and one nutritionist. The team members receive management training and should familiarize themselves with each other's roles and cooperation methods in the MDT intervention process. A WeChat group is established for the MDT intervention team to facilitate the sharing of patient-related information, and the doctor responsible for it leads the coordination of the team's work.
(2) Construction of Health Information Management for Infectious Non-union Patients: The head doctor and responsible nurse create health management records for patients, including general information, diagnostic information, treatment progress, past health issues, risk factors for infection, and deep vein thrombosis (DVT) risk factors.
(3) Implementation of MDT Intervention Model:

##### 2.2.3.1 Nutrition management

Nutrition assessment is conducted by a specialized nutrition nurse based on the patient's condition. Patients are assessed for the severity of their condition, nutritional status, and age and are scored accordingly. If the total score is ≥3, nutrition intervention is initiated under the guidance of a nutritionist. If the total score is <3, weekly nutrition reassessments are conducted, and if the reassessment score is ≥3, nutrition support is implemented. Depending on the patient's condition, nutrition support methods include oral, nasogastric tube, or intravenous nutrition (12). The nutritional plan is individualized based on daily energy requirements and specific nutritional needs.

##### 2.2.3.2 Postoperative specialized management

The responsible nurse monitors patients’ vital signs, laboratory results, and the management of complications during the perioperative period. If abnormalities are detected, timely communication with the doctor for necessary interventions is conducted. A specialized wound care nurse is available for bedside wound care for patients with complex wounds due to bone infection. The wound care nurse uses techniques to minimize the risk of needle tract infection when dealing with patients who have external fixators.

##### 2.2.3.3 Rehabilitation specialized management

The rehabilitation therapist provides individualized rehabilitation guidance based on the patient’s external fixation or wound irrigation and drainage. Rehabilitation exercises are conducted at the bedside, focusing on early rehabilitation of joints and muscles. The duration of rehabilitation is typically maintained at approximately 30–60 min. Early rehabilitation contributes to the prevention of complications such as joint stiffness and muscle contractures.

##### 2.2.3.4 Psychological assessment and intervention

Due to the potential for more extended hospitalizations, patients may experience psychological issues. The responsible doctor arranges for a psychological counselor to communicate with patients before and after surgery, with sessions lasting approximately 15 min. This helps in understanding and adjusting the patient’s psychological state. For patients with psychological issues, daily interventions are conducted under the guidance of the counselor.

##### 2.2.3.5 Summary before discharge

Discharge criteria are determined by the responsible doctor. The MDT intervention team of physicians summarizes the patient's recovery status and provides additional information such as follow-up schedules, medication guidance, and post-discharge instructions. The rehabilitation therapist creates an outpatient exercise plan based on the patient's progress and guides family members to assist in home rehabilitation. The psychological counselor summarizes the patient's current psychological state and potential emotional changes after discharge, with strategies for addressing adverse conditions and enhancing the family's psychological support skills. The nurse is responsible for information consolidation, data entry, and summarizing the guidance provided by various doctors. After that, the nurse communicates with the patient, and the wound care nurse guides wound management after discharge.

##### 2.2.3.6 Post-discharge follow-up

The responsible nurse conducts follow-up calls via phone or WeChat with the patient one week after discharge to assess their recovery progress and inquire about any rehabilitation, diet, or psychology-related issues. Any identified issues can be addressed by leaving messages in the group, and professional personnel provide solutions. Additionally, the responsible nurse periodically sends informative videos and articles to patients to assist in adjusting their post-discharge lifestyle.

### 2.3 Comparison of the two groups regarding wound infection, grade II and above pin tract infection, delayed bone healing, deep vein thrombosis, joint stiffness, and nutrition-related indicators

#### 2.3.1 Criteria for diagnosing fracture wound infection

Clinical manifestations typically include redness, swelling, heat, and tenderness around the surgical site or the local area. Pus-like fluid discharge, fistulas, and sinuses may be observed. Systemic infection symptoms such as fever and chills may also occur. Radiological examinations may reveal bone resorption, loosening of internal implants, formation of dead bone, delayed fracture healing, or non-union. Blood biochemical markers show a secondary or sustained increase in inflammatory indicators (13).

#### 2.3.2 Grading criteria for pin tract infection

Pin tract infection is assessed according to the Checketts & Otterburn pin tract infection grading criteria (14):

Grade I: Slight redness and minimal discharge around the pin tract, requiring pin tract care.
Grade II: Skin redness, discharge, and pain at the pin tract, requiring intensified pin site care and short-term antibiotic use.
Grade III: Symptoms similar to Grade II, but infection cannot be controlled with pin tract care and short-term antibiotics.
Grade IV: Severe soft tissue infection, pin loosening, and the need for pin removal.
Grade V: Besides severe soft tissue infection, x-rays show signs of osteomyelitis requiring pin removal.
Grade VI: Severe bone and soft tissue infection, requiring pin removal and incision drainage.

#### 2.3.3 Criteria for delayed fracture healing

Delayed fracture healing is diagnosed when a fracture has been treated, the fixation period exceeds the maximum required for healing a similar fracture, and the bone callus cannot firmly connect the fracture ends. There is noticeable abnormal mobility at the fracture site during limb movement, localized pain and tenderness, irregular edges of the fracture ends on x-rays, blurriness or even cystic changes, osteoporosis, and reduced bone callus growth, widening of the fracture gap, but no signs of sclerosis or marrow cavity obstruction (15).

#### 2.3.4 Criteria for joint stiffness

Western medicine diagnostic criteria are based on the diagnosis and treatment plan for knee joint stiffness developed by the National Administration of Traditional Chinese Medicine's 24 Specialties and 105 Diseases. The criteria for diagnosing joint stiffness are as follows:

The affected knee joint is stiff, not flexible, and has limited flexion and extension function, affecting daily activities such as walking and squatting.

Reduced joint range of motion compared to the healthy side. Limited flexion indicates extension-type stiffness, limited extension indicates flexion-type stiffness, and limitations in flexion and extension indicate mixed-type stiffness (16). Any limitations in joint flexion or extension are considered joint stiffness.

### 2.4 Observation indicators

### 2.5 Statistical analysis

Statistical analysis was performed using the SPSS software (version 26, IBM Chicago, IL, USA) for data entry and statistical analysis. The Shapiro-Wilk test was applied to assess the normality of distribution for continuous data in both groups. Data conforming to a normal distribution are described as (X ± s), and the two independent sample *t*-test was used for comparison. Count data are described as *n* (%), and comparisons were made using the chi-square test. For the measurements of albumin, prealbumin, hemoglobin, and serum sodium at different time points, repeated measures analysis of variance (ANOVA) was employed. For indicators showing interaction effects, separate effect analysis was conducted, and for those without interaction effects, the main effect analysis was performed. Pairwise comparisons between time points were conducted using the LSD method. The significance level for testing was set at α = 0.05, and a *P*-value < 0.05 was considered statistically significant.

## 3 Results

### 3.1 Comparison of baseline data between the two groups

There was no statistically significant difference in baseline data between the two groups (*P* > 0.05), as shown in Table 1, indicating comparability.

**Table 1:** Comparison of baseline data between the two groups [x ± s, *n* (%)].

Variable	Group		Statistic	*P*
	Study group (*n* = 40)	Control group (*n* = 40)		
Gender, *n*(%)			0.238	0.625
Male	27 (67.50)	29 (72.50)		
Famale	13 (32.50)	11 (27.50)		
Age(x ± s, 岁)	34.08 ± 7.50	33.82 ± 7.58		
Fracture site, *n*(%)			0.204	0.651
Tibia	22 (55.00)	24 (60.00)		
Femur	18 (45.00)	16 (40.00)		
Fracture type, n(%)			0.202	0.653
Open fracture	23 (57.50)	17 (42.50)		
Closed fracture	21 (52.50)	19 (47.50)		
Soft tissue condition, *n* (%)			*χ*² = 0.061	0.97
Intact	20 (50.00)	21 (52.50)		
Flap scar	6 (15.00)	6 (15.00)		
Sinus tract	14 (35.00)	13 (32.50)		
Preoperative diabetes mellitus			*χ*² = 0.287	0.592
No	32 (80.00)	30 (75.00)		
Yes	8 (20.00)	10 (25.00)		
Preoperative hypertension			*χ*²=0.051	0.822
No	18 (45.00)	17 (42.50)		
Yes	22 (55.00)	23 (57.50)		
Preoperative coronary heart disease			*χ*² = 0.251	0.617
No	30 (75.00)	28 (70.00)		
Yes	10 (25.00)	12 (30.00)		
Preoperative rheumatoid arthritis			*χ*² = 0.626	0.429
No	38 (95.00)	35 (87.50)		
Yes	2 (5.00)	5 (12.50)		

### 3.2 Comparison of infection rates between the two groups

The observation group had significantly lower rates of wound infection and level II or above pin tract infection than the control group (*P* < 0.05), as shown in Table 2.

**Table 2:** Comparison of wound infection rate, level II or above pin tract infection rate, delayed bone healing, DVT, and joint stiffness complications [cases (%)].

Group	Number	Wound infection	Level II or above pin tract infection	Delayed bone healing	DVT (Deep vein thrombosis)	Joint stiffness
Study Group	40	2 (5.00)	1 (2.50)	2 (5.00)	0 (0.00)	2 (2.50)
Control Group	40	8 (20.00)	9 (22.50)	11 (27.50)	6 (15.00)	9 (22.50)
Statistic		4.114	7.314	7.439	–	5.164
*P*		0.042	0.006	0.006	0.033	0.023

### 3.3 Comparison of nutritional indicators between the two groups after intervention

After intervention, the levels of ALB (albumin), Hb (hemoglobin), and PA (prealbumin) in the observation group were higher than those in the control group, with statistical significance (*P* < 0.05), as shown in Table 3.

**Table 3:** Comparison of nutritional indicators between the two groups after intervention.

Group	Weight		Albumin(ALB)		Prealbumin(PA)		Hemoglobin(Hb)		Serum sodium(Na)	
	Control group	Study group	Control group	Study group	Control group	Study group	Control group	Study group	Control group	Study group
Admission at 24 h	79.82 ± 7.78	81.72 ± 6.69	30.21 ± 2.40	30.19 ± 1.94	193.15 ± 14.50	190.75 ± 16.52	101.18 ± 8.12	102.19 ± 9.23	130.56 ± 3.02	131.11 ± 2.79
Pre-surgery	–	–	32.26 ± 2.26	31.89 ± 2.21	210.24 ± 25.49*	229.69 ± 39.97	103.61 ± 8.81	105.56 ± 9.50	132.20 ± 2.69	132.08 ± 3.04
Post-surgery 3 days	–	–	33.19 ± 2.57*	35.88 ± 1.87	227.87 ± 41.14*	299.28 ± 52.08	106.78 ± 8.01*	110.97 ± 6.40	132.97 ± 2.14	133.78 ± 2.64
Post-surgery 7 days	–	–	34.61 ± 2.02*	38.31 ± 1.57	241.45 ± 40.96*	343.92 ± 37.47	109.54 ± 7.79	112.05 ± 6.58	134.36 ± 1.95*	135.51 ± 2.88
Post-surgery 14 days(before discharge)	81.21 ± 7.67	83.10 ± 6.24	35.88 ± 1.61*	40.51 ± 2.41	265.06 ± 45.12*	379.79 ± 30.89	109.10 ± 8.90	111.22 ± 17.36	135.95 ± 2.36	136.51 ± 2.81
Time effect	F = 36.203 *P* < 0.001		F = 289.230 *P* < 0.001		F = 256.772 *P* < 0.001		F = 17.484 *P* < 0.001		F = 103.907 *P* < 0.001	
Group effect	F = 1.402 *P* = 0.207		F = 40.838 *P* < 0.001		F = 116.927 *P* < 0.001		F = 3.150 *P* = 0.080		F = 1.617 *P* = 0.207	
Interaction effect	F = 0.001 *P* = 0.982		F = 34.543 *P* < 0.001		F = 59.984 *P* < 0.001		F = 0.381 *P* = 0.756		F = 1.212 *P* = 0.305	

* Indicates statistically significant comparison with the control group.

## 4 Discussion

In treating infective nonunion, there are significant challenges and multiple complex issues during the perioperative period. Bone defects caused by fractures or diseases typically do not heal spontaneously, and complications and reoperation rates influence the clinical outcome. Common causes of nonunion and bone defects include open fractures, soft tissue or bone tissue loss, postoperative infections after internal fixation, acute and chronic osteomyelitis, bone tumors, and more. The rate of bone infection following severe trauma has been increasing year by year, especially with open fractures, where the infection rate can reach up to 23%. Local infections significantly reduce the bone healing rate, and controlling the infection is the primary task in treating infective bone defects (17). Current methods for treating infective nonunion include thorough debridement, local stability, dead space-filling, adequate drainage, effective coverage, antibiotic application, the Masquelet technique, and the Ilizarov bone transport technique. Patients using external fixators may experience complications during treatment, including pin tract infection, pain, joint contracture, mainly Achilles tendon contracture, ankle cartilage damage, ankle joint stiffness, dislocation, etc (18).

Furthermore, a prolonged course of the disease can bring a psychological burden to patients and pose a hidden risk to their mental health, necessitating psychological intervention. Most infective nonunion patients also suffer from malnutrition, resulting in slow wound and bone healing, necessitating assessment and intervention support from a nutritionist. The placement of Ilizarov external fixators, due to their extensive configuration, complex assembly, and inconvenience in daily carrying (19), restricts limb movement and can lead to poor venous blood circulation, making patients prone to deep vein thrombosis and adjacent joint contracture. In this study, nine patients developed joint contractures in the control group; 11 had delayed bone healing, nine had pin tract infections at level II or above, and 8 had wound infections. These are all critical and challenging issues during the perioperative period of this condition.

Multidisciplinary integrated management addresses multiple issues during the perioperative period and tailors treatment measures to individual patients in a multidimensional manner. MDT originated in the late 1990s and emphasizes the collaboration of members from different professional disciplines to provide a more comprehensive assessment and nursing guidance for clinical patients. It effectively avoids the limitations of a single profession in the design of nursing methods and has been applied in the care of patients with traumatic brain injury and cirrhosis (20, 21). In this study, after the perioperative integrated intervention, the infection rate of wounds and the infection rate of pin tracts at level II or above in the research group were lower than those in the control group. This suggests the MDT intervention model applies to patients with infective bone defects. This intervention model integrates orthopedic physicians, responsible nurses, and other professional staff members into an MDT intervention team, effectively integrating medical resources. Before and after surgery, a psychological counselor assesses the patient's inner state remotely and formulates a more professional and personalized psychological intervention plan. In combination with departmental doctors and nurses, daily psychological interventions are carried out, improving the patient's acceptance of subsequent treatment and nursing work. After surgery, specialized nursing, and rehabilitation, particular management interventions are applied, with responsible nurses focusing on monitoring and nursing the skin around the patient's wound and pin tracts. The presence of specialized dressing nurses helps avoid healthcare-associated infections caused by frequent dressing changes. For complex wounds, especially needle tracts of external fixators, individual interventions help control pin tract infections. Nutritional support by nutritionists and individualized rehabilitation exercises by rehabilitation therapists contribute to improving the patient's nutritional status and promoting wound healing. Bedside rehabilitation accompanied by guidance helps enhance the gradual recovery of joint and muscle functions for patients, especially those with frames or tubes. Many integrated management measures are coordinated by responsible doctors, and the responsible parties are clearly defined, with division of labor and cooperation playing a joint role, effectively controlling the wound infection, nutritional status, and functional recovery of patients during the perioperative period.

In summary, implementing MDT perioperative intervention for patients with infective nonunion helps control wound infections, improves the patient's nutritional status, and effectively reduces complications. This study indicates that MDT perioperative intervention for patients with infective bone defects has significant clinical value. However, insufficient medical and nursing staff allocation, especially nutritionists, rehabilitation therapists, and psychological counselors, makes it difficult to popularize the integrated management model thoroughly. In developing high-quality public hospitals in the country, integrated collaborative management will become a trend, providing patients with comprehensive services in all dimensions.

## Data Availability

The original contributions presented in the study are included in the article/Supplementary Material, further inquiries can be directed to the corresponding authors.
